# Machine learning framework for forecasting air pollution: Evaluating seasonal and climatic influences in Istanbul, Turkey

**DOI:** 10.1371/journal.pone.0330716

**Published:** 2025-10-13

**Authors:** Nadia AL-Rousan, Hazem Al-Najjar, Ismail A. Elhaty

**Affiliations:** 1 School of Computing, Computer Engineering Department, German Jordanian University, Amman, Jordan; 2 Computer Engineering Department, Faculty of Engineering, Al-Balqa Applied University, Al-Salt, Jordan; 3 Department of Nutrition and Dietetics, Faculty of Health Sciences, Istanbul Gelisim University, Istanbul, Turkey; UCSI University Kuala Lumpur Campus: UCSI University, MALAYSIA

## Abstract

Air pollution, driven by seasonal and meteorological variations, poses a significant threat to public health and urban sustainability. Despite numerous forecasting approaches, the influence of seasonal patterns on air pollutant levels remains underexplored. This study presents a computational framework utilizing the Nonlinear Autoregressive network with Exogenous inputs (NARX) model to predict concentrations of key pollutants SO₂, PM₁₀, NO, NOX, and O₃ in Esenyurt, one of the most industrialized districts in Istanbul, Turkey. Through systematic feature selection techniques, the study determines the most influential seasonal factors for each pollutant, reducing model complexity while improving predictive accuracy. The developed framework exhibits substantial improvements in predictive performance, with the optimal models achieving high determination coefficients (up to R² = 0.965 for O₃) and low error metrics across training and validation datasets. Particularly, the inclusion of seasonal variables considerably improved prediction accuracy for NO, NO₂, and PM₁₀, while SO₂ predictions performed best when utilizing comprehensive seasonal indicators. These results demonstrate that seasonal dynamics play a crucial role in governing pollutant behavior and highlight the importance of incorporating such variables in forecasting models. This research contributes significantly to the field by advancing methodological approaches in air quality prediction while providing an adaptable model for policymakers and environmental agencies to implement in proactive pollution management strategies. Through examination of seasonal dependencies in air pollutant patterns, the study delivers a practical tool for urban planning and public health applications in rapidly expanding metropolitan regions.

## 1. Introduction

Air pollution increases with the increasing of industrialization and urbanization. Public health and economic development in metropolitan cities are affected by air pollution. Air pollutants’ levels and types vary from place to another based on the sources of air pollutants such as cars, power plants, oil refineries, industrial facilities, and factories [1–3]. Air quality is monitored using the most common air pollutants (indicators) including SO₂, NO₂, CO₂, O₃, NO, NOₓ, PM2.5, and PM10 [4–6]. These indicators can be found in different levels in the ambient air, unfortunately, exceeding the concentration levels of these pollutants will threaten human health and may cause many serious problems, namely long term and short-term problems. One of the most hazardous events produced by air pollutants is the great smog of 1952 in London, which continued for five days and killed 4000. Monitoring and detecting the concentration of air pollutants can help decision makers to take the right decisions for the current and future plans. An enormous amount of research have been conducted to forecast the pollutants’ concentrations and to understand the most suitable way to evaluate the air quality.

Cogliani [7] studied the relationship between metrological variables and daily pollution index in three Italian cities using linear multiple partial correlation analysis. The results of forecasting the concentration of pollutants showed high evaluation error and the methods can be used in the surrounding areas of the observation stations while forecasting the pollutants in places that far away from the station is inapplicable.

Zhu et al. [8] investigated the relationship between low respiratory diseases and monthly average concentration of SO_2_, NO_2_ and PM_10_ by considering the effect of seasons especially winter season. Besides, the study tried to estimate the dataset covered the period from January 2001 to December 2005 and was collected from Xigu District’s hospitals. The results found a relationship between short-term pollution and low respiratory diseases and found strong relation with winter season on low respiratory diseases. Feng et al. [9] proposed a novel daily PM_2.5_ forecasting to improve the performance of artificial neural network by using air mass trajectory analysis and wavelet transformation. The dataset is collected from 13 stations from different locations in China including Beijing, Tianjin, and Hebei provinces. The results showed that the new hybrid method can reduce the root mean square error up to 40%. Furthermore, the results indicated that the proposed model is efficient to be applied in different countries.

Fortelli et al. [10] investigated the relationship between local metrological variables and PM10 in Naple, IItaly. Afterward, metrological variables are used to forecast PM_10_ for couple of days. The results found a relationship between air pollution crises and geopotential heights. The prediction model showed a high correlation between PM_10_ observations and the predicted values with 0.8 as a correlation coefficient. Alimissis et al. [11] evaluated two interpolation prediction models including Artificial Neural Networks and Multiple Linear Regression, to predict the quality of air in Athens, Greece. The quality of air is majored using five pollutants including Nitrogen dioxide, Nitrogen monoxide, Ozone, Carbon monoxide and Sulphur dioxide. The results showed that artificial neural networks are found in most cases to be significantly superior, especially where the air quality network density is limited.

Yu et al. [12] proposed a fast-forecasting method to estimate PM_2.5_ concentrations in six cities including Baoding, Beijing, Dezhou, Shijiazhuang, Tianjin, and Tangshan, which located in the north of China. The forecasting method is based on source–receptor relationship modeling with backward Lagrangian stochastic particle dispersion model and emission inventory inversion. The forecasting method is built using a dataset collected in 2015, where another dataset collected in 2016 is used for forecasting purposes. The results showed that applying the new techniques can achieve better results and high correlation coefficients compared with non-optimized models. Wang et al. [13] developed forecasting model to predict an interval PM2.5 concentration using meteorological factors based on multilayer perceptron. To select the most important input variables from the list of variables an interval grey incidence analysis is adopted. The dataset is collected from three locations in Beijing, China. The developed model showed more accurate and stabile results compared to other models in the literatures. Liu et al. [14] developed a three stages hybrid algorithm based on neural network to forecast PM_2.5_. The dataset is collected from four different cities in China namely, Beijing, Tianjin, Shijiazhuang, and Tangshan. The results revealed that the proposed model is efficient compared to conventional methods.

Lu et al. [15] investigated the relationship between the two pollutants PM_2.5_ and PM_10_ in different locations within Hong Kong province. A predictive model is employed to estimate the concentration of PM_2.5_ using PM_10_. The results showed the ability to estimate the missing and unmonitored values. Zhu et al., [16] proposed a hybrid prediction model to estimate concentrations of NO_2_ and SO_2_ pollutants in four cities in China. The model is divided into three steps starting with finding high frequency and low frequency sequences, followed by applying Support Vector Regression based on combining the Cuckoo Search algorithm and Grey Wolf Optimizer algorithm and finally, forecasting data of low and high frequency.

Catalano et al. [17] studied the relationship between the hourly mean concentration of NO_2_ and the factors that reflect the NO_2_ level (i.e., traffic and weather conditions). Both neural network and Autoregressive Integrated Moving Average with Explanatory (ARIMAX) forecasting methods were used to predict the pollution peaks along with using a combination of these models to forecast the air quality. The results revealed that ARIMAX outperformed neural network in pollution peak forecasting, while neural network could better represent the realistic pollution’s concentration association with wind attribute. Integrating both forecasting models could efficiently predict extreme pollution concentrations than using both models separately.

Durao et al. [18] forecasted the concentration level of O_3_ using a combination of metrological and air quality and industrial emissions data for Sines Portuguese region, Portugal. Both Multi-Layer Perceptron (MLP) and Classification and Regression Trees (CART) models were employed to predict O_3_ concentration. The models could successfully predict O_3_ concentration within 24 hours ahead.

Corani et al. [19] proposed a multi-label classifier that can predict multiple air pollutants. Bayesian networks were employed to predict the level of PM_2.5_ and ozone. It is found that using multi-label classifier performed better than other classifiers.

Choudhary et al. [20] developed a time series ARIMA model to forecast monthly concentrations of PM2.5, NO₂, O₃, and MODIS-derived AOD over an urban traffic site in New Delhi. The study employed data from 2012 to 2017 to train the model, and data from 2018–2019 to validate the results. The results revealed that pollutant concentrations, particularly PM2.5, were significantly higher than the national standards. The ARIMA model achieved high accuracy in forecasting with stationary R² up to 0.752 for PM2.5 and consistent trends across pollutants. The study revealed that ARIMA is an effective tool for simulating and forecasting urban air quality.

Choudhary et al. [21] predicted the distribution of criteria pollutants (PM2.5, PM10, NO₂, SO₂, CO, and O₃) in the industrial belt of eastern coastal India using Random Forest (RF), Support Vector Machine (SVM), Bagged Multivariate Adaptive Regression Splines (MARS), and Bayesian Regularized Neural Networks (BRNN). The study used air quality data from Talcher and Brajrajnagar between January 2019 and June 2021. Results found that RF, SVM, bagged MARS, and BRNN achieved strong correlations (r = 0.83–0.92) and low RMSE values (e.g., PM2.5 RMSE as low as 12.40) for most pollutants, except for CO and O₃ which showed weaker predictive performance.

Sharma et al. [22] investigated the forecasting of PM2.5 concentrations across selected satellite cities surrounding Delhi using the ARIMA model. ARIMA was employed to forecast PM_2.5_ concentrations and was validated using ground-station data. Results found a strong predictive accuracy with R² values above 0.82 and RMSE as low as 18.28. The model outperformed other models concluded that ARIMA is a robust method for PM_2.5_ forecasting.

Several studies have explored the impact of meteorological factors on air quality. Shi et al. [23] found that PM_2.5_ level in Central East China were significantly influenced by wind direction and radiative cooling. Similarly, Mo et al. [24] developed a hybrid machine learning model to predict surface ozone concentrations across four Chinese stations, demonstrating high accuracy and transferability. Several researchers have widely applied machine learning [25,26], autoregression models [27], and hybrid approaches to forecast air quality indicators [28–30].

However, pollutants can cause acid rain that has a harmful effect on buildings, plants, monuments, soil composition, groundwater, and aquatic life. Several studies that have used multiple linear regression (MLR), artificial neural networks (ANN), and hybrid forecasting methods suffer from either overlooking the nonlinear nature of pollution dynamics or failing to incorporate time lagged variables and seasonality, thus, causing the limitation in their accuracy and generalization. To address these issues, this study adopts the NARX model, which integrates both nonlinear modeling capability and memory of past values through autoregressive feedback. This makes it particularly effective in capturing the seasonal patterns and delayed interactions commonly observed in air quality data. Thus, this study is important as it gives an opportunity for decision-makers to take into account the level of air pollution when developing future plans especially at a time when Turkey is aiming to increase exports, attract investments, and expand construction of factories. Despite numerous studies, few have focused on the seasonal effects on pollutant trends, particularly concerning key pollutants like SO₂, NO, NO₂, NOX, O₃, and PM10. Furthermore, many researchers have explored different combinations of input variables without relying on scientific or mathematical justification.

**Fig 1:**
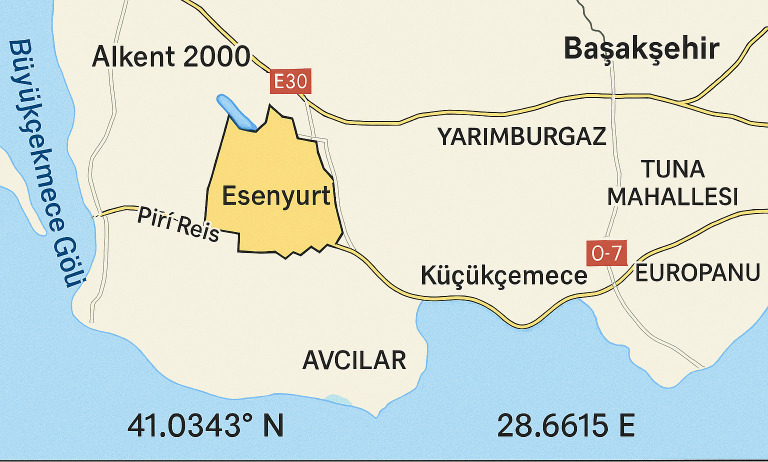
Map highlighting Esenyurt district in Istanbul, Turkey.

Therefore, this article presents essential findings for both international and national researchers. This study was conducted in Esenyurt, a district of Istanbul Province that belongs to a metropolitan municipality of the city (41.0343° N, 28.6615° E). Esenyurt is situated in the European part of the city and km far from Marmara Sea. it’s surrounded by Avgelar (Avcılar) district and Lake Kochokmaje (Küçükçekmece four) on the eastern part; Buyukchakmaje (Büyükçekmece) on the western region; Bashakshaheer (Başakşehir), Arna’outkoy (Arnavutköy) and Trans-European Motorway (TEM) on the northern region; and Bailekdazou (Beylikdüzü) and E-5 motorway on the southern portion. Its municipality has existed since 1989 and its total area is 43.0 km2. The construction and population are growing rapidly during the last decade. The population estimate is 954579 in 2019 with population density 22.20/km2 (State Institute of Statistics, Republic of Turkey). Fig 1. shows the geographical location of Esenyurt within the Istanbul province, highlighting the area where data for this study was collected.

Our contributions to this research are explained as follows:

- There is a lack of studies considering the impact of seasons in understanding the trend of pollutants in air.
- Very few studies apply feature selection with seasons and pollutants to determine the most effective season(s) for each pollutant.
- This study introduces a new methodology that improves the forecasting of pollutants including SO₂, NO, NO₂, NOX, O₃, and PM10. It also reveals the behavior of each independent variable concerning each pollutant.
- The research identifies the most effective season(s) for predicting major pollutants in Turkey, providing a model that can be adapted to other regions.
- It demonstrates the forecasting capability of the Nonlinear Autoregressive network with Exogenous inputs (NARX) model for different pollutants, offering a valuable and efficient alternative to other models.

Major findings from the results validate the importance of this approach. Notably, the study shows that including seasonal variables significantly improves forecasting performance. For example, PM10 predictions were most accurate with spring data, while O₃ and NO₂ showed optimal results with autumn and spring inputs. SO₂ and NO forecasts improved when all seasonal factors were considered. The NARX model consistently delivered high accuracy, with R² values reaching 0.965 in some cases, proving its robustness and reliability.

This study contributes a scientifically grounded, season-sensitive forecasting model that supports effective air quality management and policymaking. It demonstrates that addressing seasonal variations is not only beneficial but essential for achieving accurate predictions of urban air pollution levels.

## 2. Research methodology

To develop a predictive model that aims to forecast gases concentrations in air, a systematic feature selection methodology was employed to identify correlations between seasonal variables and pollutants as shown in the following subsections.

### 2.1 Data collection and analysis

This study was conducted in Esenyurt, a polluted and rapidly urbanizing districts of Istanbul, Turkey. The dataset spans a 5 year period starting from 2015 to 2019. The dataset comprises 37,645 hourly records of air pollutant concentrations and meteorological variables. The data were obtained from the National Air Quality Monitoring Network managed by the Ministry of Environment, Urbanization, and Climate Change of Turkey, and are publicly accessible [31]. A copy of the dataset used in this study is provided as S1 Dataset.

The primary pollutants monitored in this study include sulfur dioxide (SO₂), nitrogen oxide (NO), nitrogen dioxide (NO₂), nitrogen oxides (NOₓ), ozone (O₃), and particulate matter (PM10). Meteorological variables such as temperature, wind speed, humidity, and atmospheric pressure were also collected from the same location to support a holistic forecasting approach.

### 2.2 Permits and ethical approval

In this study, no specific permits were required, as it utilized publicly accessible air quality and meteorological data from the Turkish Ministry of Environment, Urbanization, and Climate Change via the National Air Quality Monitoring Network. The research did not involve field site access, specimen collection, or human participants, and therefore did not require ethical approval or special permissions.

### 2.3 Data preprocessing

To prepare the dataset for machine learning modeling, the following preprocessing steps were applied:

- Missing Data Treatment: Short gaps in the time series were addressed using linear interpolation, while long gaps (exceeding 10% of the total records) were removed to maintain data quality.
- Normalization: All numerical features were normalized using Min-Max scaling to standardize the input range between 0 and 1.
- Seasonal Classification: Each data record was classified by season (Spring, Summer, Autumn, Winter) according to its temporal occurrence. This would enable the examination of seasonal influence on pollutant concentrations.
- Feature Selection: Statistical correlation analysis was conducted to identify and retain the most influential input variables for forecasting each pollutant.

### 2.4 Model selection

Given the nonlinear dynamics and temporal dependencies of air pollution data, the Nonlinear Autoregressive model with Exogenous Inputs (NARX) was selected. NARX models incorporate feedback loops and time-lagged dependencies, making them well-suited for capturing complex patterns in time-series data. Unlike traditional models like Multiple Linear Regression (MLR) or conventional neural networks, the NARX model excels in forecasting scenarios where both seasonal variation and delayed input-output relationships are significant. This selection is also supported by recent literature demonstrating NARX’s superior performance in environmental prediction applications.

### 2.5 Model configuration

The dataset was partitioned chronologically into two segments: Training set (2015–2018) and Testing set (2019). This temporal division follows an 80:20 ratio, ensuring the model learns from historical patterns and validates performance on prospective data. Model optimization was conducted using the Levenberg–Marquardt (trainlm) algorithm. This methodology ensures reliable model performance and supports accurate forecasting under actual urban pollution conditions. Table 1 presents a statistical summary of training and testing datasets, including descriptive statistics such as minimum, maximum, mean, and standard deviation for key air pollutants (SO₂, NO, NO₂, NOₓ, O₃, PM₁₀) and meteorological parameters. These statistics demonstrate the range and variability of environmental conditions in Esenyurt during the study period and provide essential context for understanding pollutant behavior and distribution patterns. The observed concentration peaks and seasonal fluctuations support the selection of a dynamic nonlinear forecasting approach such as NARX.

**Table 1:** Statistical analysis of training and testing datasets.

Variable	Mean		Error		Standard Deviation		Variance		Minimum		Maximum	
	Training	Testing	Training	Testing	Training	Testing	Training	Testing	Training	Testing	Training	Testing
Time	0.482	0.486	0.002	0.004	0.286	0.289	0.082	0.083	0	0	0.958	1
Day	15.734	15.634	0.05	0.104	8.827	8.602	78	73.999	1	1	31	31
Month	6.476	5.834	0.02	0.038	3.436	3.148	12	9.911	1	1	12	12
Year	2016	2019	0	0	1.099	0	1	0	2015	2019	2018	2019
Temperature	16.244	17.509	0.045	0.094	7.886	7.756	62	60.152	−5	0.12	38	34
Wind direction	195.34	192.874	0.551	0.921	96.682	75.97	9347	5771.462	0	34.86	360	350
Wind speed (ms^-1^)	2.225	2.11	0.006	0.012	1.038	1.021	1	1.043	0	0.06	7	6
Relative humidity(%)	73.645	73.382	0.093	0.198	16.336	16.304	267	265.829	0	16.08	100	100
Air pressure (mbar)	1011.94	1010.477	0.06	0.068	10.64	5.573	113	31.06	0	993.39	1036	1028
Summer	0.257	0.273	0.002	0.005	0.437	0.446	0	0.199	0	0	1	1
Winter	0.243	0.201	0.002	0.005	0.429	0.401	0	0.161	0	0	1	1
Spring	0.259	0.305	0.002	0.006	0.438	0.46	0	0.212	0	0	1	1
Autumn	0.241	0.221	0.002	0.005	0.428	0.415	0	0.172	0	0	1	1
PM10 (µgm-³)	81	60	0	1	78	42	6013	1801	0	6	985	509
SO2 (µgm-³)	6	10	0	0	7	7	46	51	0	0	220	100
NO (µgm-³)	43	20	0	1	71	45	5047	2033	0	0	880	696
NO2 (µgm-³)	24	29	0	0	17	18	281	332	0	2	130	152
NOX (µgm-³)	90	61	1	1	116	82	13390	6760	0	2	1338	1219
O3 (µgm-³)	39	24	0	0	26	17	692	292	0	0	866	97

To ensure obtaining an effective learning and reliable convergence in the NARX model, standard hyperparameters from the MATLAB MathWorks Neural Network Time Series Toolbox were employed. Two hidden layers were employed with 20 and 10 neurons respectively. To support continuous pollutant prediction, a hyperbolic tangent sigmoid (tansig) activation function was used in the hidden layers, while a linear (purelin) activation function was used in the output layer. The model was trained using the Levenberg–Marquardt (trainlm) optimization algorithm with a maximum of 1000 epochs and early stopping based on validation performance. The main loss function used was Mean Squared Error (MSE), which is standard for time series regression tasks. Table 2 summarizes the hyperparameter configuration adopted for model training. These choices reflect MATLAB’s default NARX setup and have proven effective for nonlinear time-series forecasting in environmental contexts.

**Table 2:** NARX model configuration hyperparameters.

Parameter	Value
Network Type	NARX (Nonlinear Autoregressive Network with Exogenous Inputs)
Number of Hidden Layers	2
First Hidden Layer Size	20 neurons
Second Hidden Layer Size	10 neurons
Activation Function (Hidden Layers)	tansig (Hyperbolic Tangent Sigmoid)
Activation Function (Output Layer)	purelin (Linear)
Training Algorithm	Levenberg–Marquardt (trainlm)
Number of Epochs	1000 (with early stopping)
Loss Function	Mean Squared Error (MSE), other performance metrics were used as literature
Toolbox Used	MATLAB MathWorks Neural Network Time Series Toolbox

To implement the effect of seasons in the collected dataset, four dummy variables representing each season were created. The values corresponding to seasons in the statistical tables represent binary dummy variables (0 or 1) used to indicate the presence of a specific season for each observation. For example, if the observation occurred during spring, the spring variable is set to 1, while the other seasonal variables (summer, autumn, winter) are set to 0. These binary indicators are included as features to assess the seasonal influence on pollutant concentration. The statistical description of the training and testing datasets is presented in Table 1. The input features include Time, Day, Month, Year, Temperature (°C), Wind Direction (Degree), Wind Speed (m·s ⁻ ¹), Relative Humidity (%), Air Pressure (mbar), and seasonal variables (based on feature selection results), while the output variable is one of the air pollutants: PM₁₀ (µg·m ⁻ ³), SO₂ (µg·m ⁻ ³), NO (µg·m ⁻ ³), NO₂ (µg·m ⁻ ³), NOₓ (µg·m ⁻ ³), or O₃ (µg·m ⁻ ³), depending on the modelling case.

To assess the performance of each predictive model, four statistical metrics were employed: Coefficient of Determination (R²), Mean Squared Error (MSE), Root Mean Squared Error (RMSE), and Mean Absolute Error (MAE). These metrics are widely recognized in environmental forecasting and time-series modelling literature as effective tools for evaluating the deviation between predicted and actual values [32–34].

### 2.6 Design seasons’ models based on feature selection

After collecting the data from the source, data processing should be conducted. Since the collected data has no missing value, no outlier values, therefore, sample modelling and design should be the next step to validate the relationship between independent and dependent variables, besides, to find the capability of forecasting air pollution. The study uses six type of air pollutants including PM10, SO2, NO, NO2, NOX and O3, besides Turkey has four seasons, so the total number of models that can be generated is equal to 144 models (24 “seasons’ combinations” X 6 “models”). Trying all the combinations is time consuming and a huge number of results will be generated.

**Fig 2:**
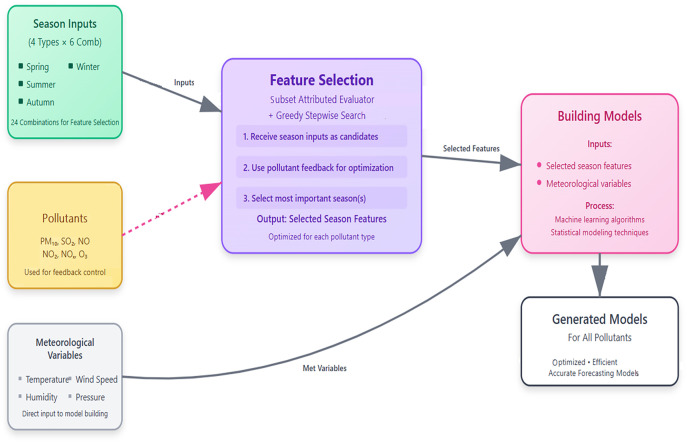
The created models’ methodology.

To minimize the total number of models, a subset attributed evaluator with greedy stepwise search method is used as a feature selection method. The target of using a feature selection is to find the most important seasons that connected with each studied pollutant. The process starts by selecting one of the pollutants as a target and all the seasons as input where the rest of metrological variables are used without using feature selection based on the previous studies. The most important season(s) for each pollutant is/are considered. The results of all the models are used to create models. The created models are used to design forecasting models for all the pollutants as shown in Fig 2.

In our dataset, a long time hourly (i.e., 30840 hourly readings) data is used to find the most related seasons that connected with each pollutant, then there will be 6806-time series samples for forecasting purposes. The most important seasons and the designed models will be discussed in the results section.

### 2.7 Air pollution gases forecasting based on NARX

After building models using feature selection and metrological variables. The NARX forecasting model will be used to forecast different pollutants. The first step in building a forecasting model is to train the NARX model using a training dataset between 2015 and 2018. The NARX will run many times until the best model is achieved. The best model that has highest determination coefficient and minimum error are considered for each pollutant. The testing dataset that is not used in the training phase between Jan 2019 to Dec 2019 is used to forecast the performance of the trained NARX. The previous two steps are repeated to find the most appropriate weights for NARX models. The best model for each pollutant is used to calculate performance metrics. For each pollutant, all the generated models are considered and the best model that achieved the best performance metrics are denoted as the best model based on testing dataset (not training dataset). The results generated from NARX and feature selection method are compared together to draw conclusion about the pollutant type and season(s).

**Fig 3:**
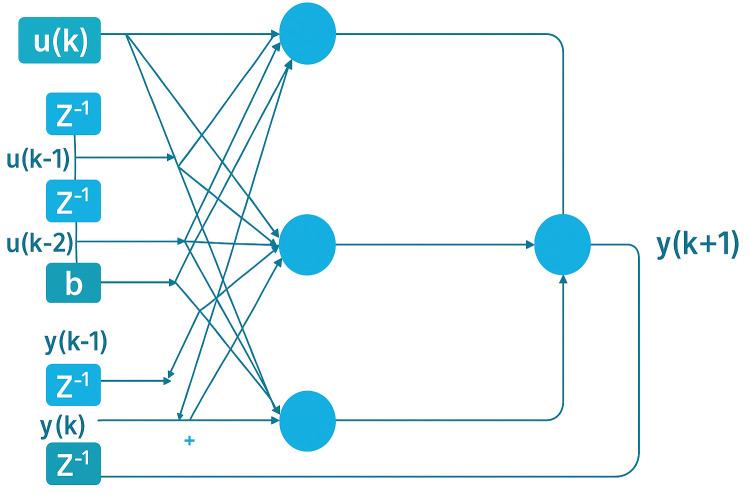
NARX model with tapped delay line at input.

Fig 3 shows the NARX network with one output (pollutant) denoted as y, u inputs and b as bias, the process of NARX starts by creating serial parallel architecture (opened loop network), then parallel architecture (closed loop network). The target of creating an open and closed network is to improve the forecasting process and to increase the efficiency of the network by using the previous direct data. To forecast the trained NARX model, the first two inputs with training data are used by forecasters to adjust the predicted values.

**Fig 4:**
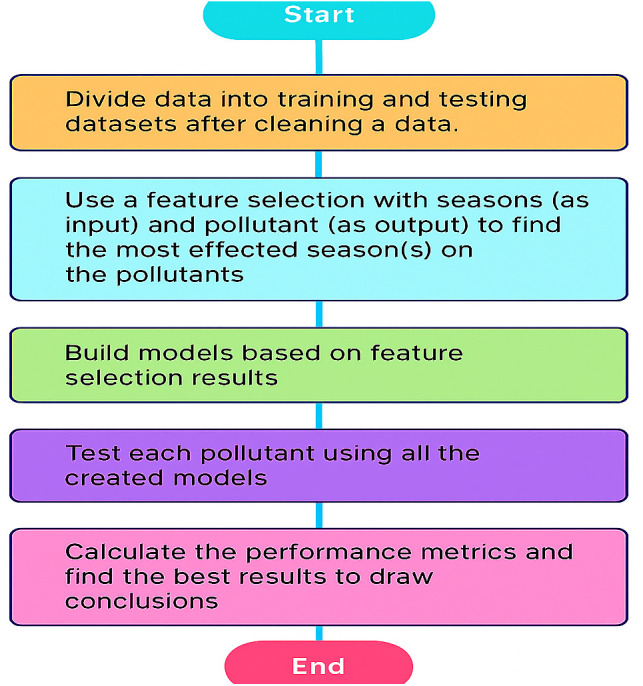
Workflow of the methodology used in this study.

To sum up, the entire workflow and methodology used to build the forecasting models are presented in the flowchart shown in Fig 4.

### 2.8 Performance analysis

After building pollutant time series model for each pollutant using a training dataset, a testing dataset is used to determine the most effective and suitable model for each pollutant. The results of training and testing datasets using different generated models are evaluated using four metrics including determination coefficient (R2), mean square error (MSE), mean absolute error (MAE) and root mean square error (RMSE).The calculations of all the performance metrics are presented as follows:


$$\;R^{\text{2}}=\text{1}-\;\frac{\sum_{i=\text{1}}^{N}{\left(y_{i}-\hat{y_{i}}\right)}^{\text{2}}}{\sum_{i=\text{1}}^{N}{\left(y_{i}-\overset{―}{y}\right)}^{\text{2}}}\;\;\;\;\;\;\;\;\;\;\;\;\;\;\;\;\;\;$$ (1)

$$RMSE=\sqrt{\frac{\text{1}}{N}\sum {\mathrm{\backslash nolimits}}_{i=\text{0}}^{N}{\left(y_{i}-\hat{y_{i}}\right)}^{\text{2}}\;}\;\;\;\;\;\;\;\;\;\;\;\;\;\;\;\;$$ (2)

$$\;MSE=\frac{\text{1}}{N}\sum {\mathrm{\backslash nolimits}}_{i=\text{0}}^{N}{\left(y_{i}-\hat{y_{i}}\right)}^{\text{2}}\;\;\;\;\;\;\;\;\;\;\;\;\;\;\;\;\;$$ (3)

$$\;MAE=\frac{\text{1}}{N}\sum {\mathrm{\backslash nolimits}}_{i=\text{0}}^{N}\left|y_{i}-\hat{y_{i}}\;\right|\;\;\;\;\;\;\;\;\;\;\;\;\;\;\;\;\;$$ (4)

The higher R^2^ and the low error function are considered as a best model, robust and accurate, besides the seasons of the best results are pollutants considered the most effected results that can explain the concentrations of the pollutant.

## 3. Results, analysis and discussion

### 3.1. Relationship between input variables and air pollutants

To show the impact of each independent variable (input) on the concentration of air pollutants including PM_10_, SO_2_, NO, NO_X_, NO_2_ and O_3_, correlation coefficients are shown in Table 3. The most effective date variable on the pollutants is the year variable, which shows a decrease in the concentration of all pollutants per year (except SO_2_).

**Table 3:** Correlation coefficient between input and output variables.

Variable	PM10	SO2	NO	NO2	NOX	O3
Time	0.110	0.082	−0.003	0.159	0.021	0.092
Day	−0.028	−0.021	−0.007	−0.004	−0.005	0.004
Month	−0.011	−0.096	0.048	−0.037	0.049	−0.043
Year	−0.226	0.190	−0.075	−0.020	−0.067	−0.050
Temperature	−0.055	−0.136	−0.254	−0.109	−0.245	0.380
Wind direction	0.057	0.045	0.197	0.094	0.198	−0.212
Wind speed	−0.225	−0.061	−0.355	−0.416	−0.395	0.441
Relative humidity	−0.071	−0.115	0.149	0.018	0.142	−0.395
Air pressure	0.074	0.025	0.130	0.051	0.130	−0.136
Summer	−0.102	−0.117	−0.205	−0.134	−0.207	0.255
Winter	−0.005	0.245	0.143	0.041	0.136	−0.187
Spring	0.116	−0.039	−0.024	0.047	−0.027	0.036
Autumn	−0.011	−0.083	0.093	0.048	0.106	−0.114

Decreasing the concentration of air pollutants (except SO_2_) with time may be attributed to the high percentage of modern cars in Istanbul which emit less pollutants compared with the old ones. In addition, such decreasing may be attributed to the rules issued by Turkish government to control the emitted pollutants from cars and factories. The growing industry in Turkey, particularly Istanbul, may interpret the increasing concentration of SO_2_ with time. In general, sulfur dioxide releases from fossil fuel burning power stations, industrial processes such as extracting metal from ore and the burning of fuels with a high sulfur content by locomotives, large ships and non-road equipment [35].

In Esenyut, the temperature ranged between −5°C in winter and 38°C in summer during the study period. Temperature has a negative correlation with all pollutants except ozone which showed a positive correlation. These results are almost consistent with the correlation between air pollutants and winter and summer. In this study, the negative correlation could be attributed to the decrease in usage of the domestic heating in summer. In the case of ozone, it’s mainly formed by a photochemical reaction consequently the more intense the solar radiation (temperature), the more O_3_ concentrations [36]. Wind speed and direction vary widely in Esenyurt. The average wind speed is 2.225 m/s during the study while the wind direction at the most is NNE. Wind direction has a positive low correlation with PM_10_, SO_2_, NO, NO_2_ and NO_X_, where O_3_ has negative intermediate relationship with wind direction. Air pollutants in Esenyurt are released mainly from local sources such as local industries, traffic and domestic heating. Wind transport air pollutants from Esenyurt to the surrounding places or the inverse. Positive correlation indicates that wind may transport air pollutants towards the monitoring sites. Ozone is unstable molecule, and it may be affected by the air movement. In general, it has been found that ozone concentration is higher in the places surrounding the city than inside the city center [37].

In general, as the humidity increases, the concentration of air pollutants decreases because of the washing effect [38]. The interference between relative humidity and other parameters such as temperature explained the variety of the correlation between relative humidity and the concentration of air pollutants indicating that it’s difficult to analyze meteorological variables [39]. Air pressure showed a positive correlation with all gases except with O_3_. The positive results agree with the reported works [25]. The negative effect of pressure on ozone layers may be attributed to the depletion of ozone under pressure.

### 3.2. Designing forecasting models based on season’s variables

Before building a forecasting model and avoiding a lot of combinations between seasons, a feature selection between the season variables and each pollutant gas is considered. Table 4 represents the best effected season on each gas. Based on Table 4 and after combining the most effective seasons, 6 models could be generated as represented in Table 5. Models 1 and 6 represent no season effect and all the season effects, respectively. Model 3 represents spring and Autumn, respectively, where Models 4 and 5 represent summer-winter and Autumn-spring seasons respectively. Therefore, instead of testing 144 models only 6 models are considered in this research. Each gas from the pollution list will use all the models and the best model for each gas are recorded separately as shown in the next section.

**Table 4:** The most effect season(s) on each gas using subsets attribute evaluator with greed stepwise search method.

Gas	Most Effected Season
PM_10_	Spring
SO_2_	Summer, winter
NO	Summer, winter
NO_2_	Autumn
NOX	Autumn, Spring
O_3_	Spring, Autumn, Spring, Winter

**Table 5:** Model design and description of each model.

Model No.	Description
1	Without Season effect
2	Spring
3	Autumn
4	Summer, Winter
5	Autumn, Spring
6	Spring, Autumn, Spring, Winter

### 3.3. Air pollution gases forecasting results

Before building a forecasting model and avoiding a lot of combinations between seasons, a feature selection between the season variables and each pollutant gas is considered. Table 4 represents the best effected season on each gas. Based on Table 4 and after combining the most effective seasons, 6 models could be generated as represented in Table 5. Models 1 and 6 represent no season effect and all the season effects, respectively. Model 3 represents spring and Autumn, respectively, where Models 4 and 5 represent summer-winter and Autumn-spring seasons respectively. Therefore, instead of testing 144 models only 6 models are considered in this research. Each gas from the pollution list will use all the models and the best model for each gas are recorded separately as shown in the next section.

### 3.4. Air pollution gases forecasting results

The correlation results in Table 3 showed that autumn and spring seasons have a weak correlation with NO, which means that these two seasons may have a strong nonlinear relationship. The results of training and testing NO forecasting are shown in Table 6. The results showed that the best model for training is Model 5 with R^2^, MSE in (µg/m³)², MAE in (µg/m³) and RMSE in in (µg/m³) equal to 0.95, 508, 10. 23, respectively. While Model 6 showed that the best results for testing are achieved with R^2^, MSE, MAE and RMSE equal to 0.901, 431, 12, 21, respectively. The results indicate that Autumn and Spring seasons are efficient for training the forecasting model, where for testing, Model 6 showed better performance.

**Table 6:** The performance of NO gas prediction using training and testing datasets.

Dataset	Model	Gas	R^2^	MSE (µg/m³)²	MAE(µg/m³)	RMSE (µg/m³)
Training	1	NO	0.944	559.89	10.86	23.66
	2	NO	0.946	540.50	10.99	23.25
	3	NO	0.943	571.06	10.82	23.90
	4	NO	0.944	551.49	10.81	23.48
	5	NO	0.950	508.47	9.84	22.55
	6	NO	0.949	507.04	10.62	22.52
Testing	1	NO	0.894	430.62	10.18	20.75
	2	NO	0.864	533.95	11.18	23.11
	3	NO	0.877	565.95	14.14	23.79
	4	NO	0.890	456.10	11.22	21.36
	5	NO	0.867	540.75	10.83	23.25
	6	NO	0.901	431.54	11.59	20.77

**Fig 5:**
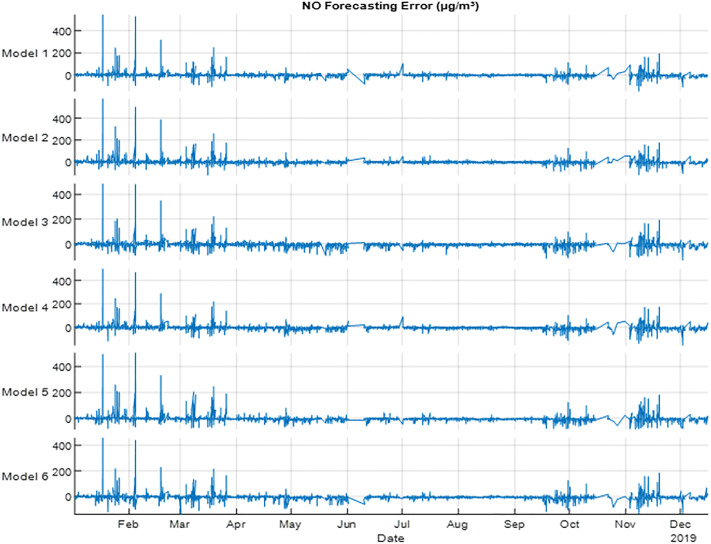
NO error (µgm-³) forecasting for 2019 using NARX.

To show the fluctuation of errors in the designed models Fig 5 showed forecasting errors of NO gas in 2019. All models showed higher errors on February and March, where the results of months showed lower errors. The results indicated that using all the seasons variables with metrological variables and time to forecast NO gas is efficient. This indicates that NO gas has a relationship with the seasons, and it change every season.

**Fig 6:**
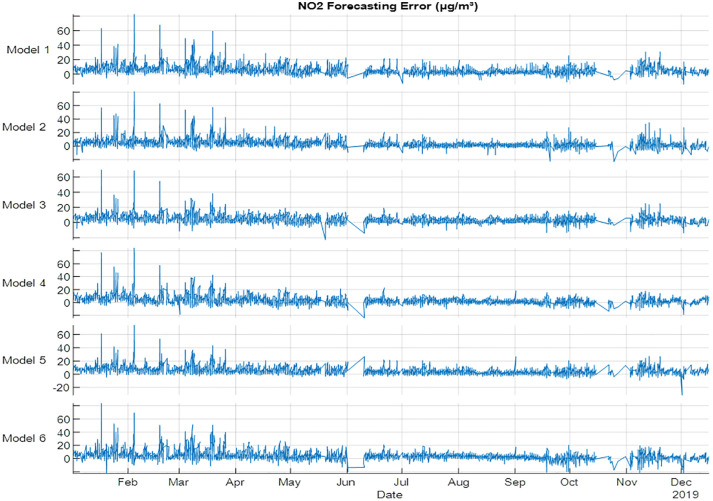
NO2 error (µg/m³) forecasting for 2019 using NARX.

The results of forecasting NO_2_ gas are shown in Fig 6 and Table 7. NO forecasting results showed that Model 5 and Model 3 are the best models for training and testing respectively. The best R^2^, MSE, MAE and RMSE are 0.959, 24, 3 and 5, respectively for training, where the best R^2^, MSE, MAE and RMSE for testing are 0.973,37, 4 and 6, respectively. The forecasting error of NO_2_ for 2019 is shown in Fig 6, the results of errors showed that February and March have the highest errors using all the models, which indicated that using the metrological variables with Autumn season could achieve the best results for training and testing datasets. The results revealed that using the season effect can improve the NO_2_ forecast.

**Table 7:** The performance of NO_2_ gas prediction using training and testing datasets.

Dataset	Model	Gas	R^2^	MSE (µg/m³)²	MAE (µg/m³)	RMSE(µg/m³)
Training	1	NO_2_	0.945	30.81	3.73	5.55
	2	NO_2_	0.910	48.93	4.79	7.00
	3	NO_2_	0.958	24.29	3.47	4.93
	4	NO_2_	0.949	29.15	3.74	5.40
	5	NO_2_	0.959	24.20	3.48	4.92
	6	NO_2_	0.938	34.57	4.01	5.88
Testing	1	NO_2_	0.961	56.87	5.35	7.54
	2	NO_2_	0.960	40.44	4.14	6.36
	3	NO_2_	0.973	36.81	4.28	6.07
	4	NO_2_	0.953	44.20	4.32	6.65
	5	NO_2_	0.971	49.07	4.97	7.01
	6	NO_2_	0.950	49.06	4.48	7.00

Moreover, it was observed that in some cases, the R² value for the testing dataset slightly exceeded that of the training dataset. While this may initially appear counterintuitive, it can be attributed to the statistical properties of the testing data, which may contain more consistent trends or fewer anomalies than the training set. This phenomenon is not indicative of overfitting or model bias. On the contrary, it highlights the model’s ability to generalize well to unseen data, especially when the model is regularized and tuned adequately.

To further confirm that the model is not overfitting or biased, we performed comparative evaluations across multiple models using different seasonal splits and data partitions. In all cases, performance metrics such as RMSE and MAE remained consistent between training and testing datasets, and no model showed erratic fluctuations that would suggest memorization of training data.

Regarding the performance metrics, the units of MSE, MAE, and RMSE in all tables correspond to the same units used in measuring the concentration of NO₂ gas, which is micrograms per cubic meter (µg/m³). This ensures consistency and interpretability when comparing the magnitude of errors across all models.

**Fig 7:**
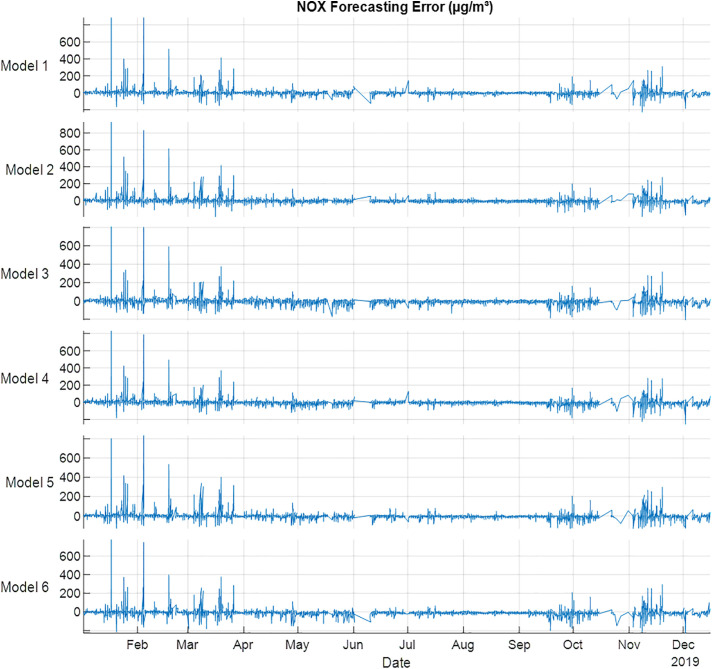
NOX error (µg/m³) forecasting errors for 2019 using NARX.

For NO_X_ training results, Model 5 showed the best performance with R^2^, MSE, MAE and RMSE are 0.951,1332, 17 and 37, respectively, where for testing results, Model 1 showed the best performance in R^2^, MSE, MAE and RMSE with values equal to 0.951, 1142, 16 and 34, respectively as shown in Table 8. Fig 7 showed the forecast errors of NO_X_ gas using Models 1–6. The forecasting results showed that February and March have the highest errors. The results indicated that no season effect is the best model to describe the behavior of NO_X_.

**Table 8:** The performance of NO_X_ gas prediction using training and testing datasets.

Dataset	Model	Gas	R^2^	MSE (µg/m³)²	MAE (µg/m³)	RMSE (µg/m³)
Training	1	NO_X_	0.946	1419.45	17.64	37.68
	2	NO_X_	0.944	1478.56	18.86	38.45
	3	NO_X_	0.942	1526.45	18.14	39.07
	4	NO_X_	0.944	1474.48	18.21	38.40
	5	NO_X_	0.951	1332.15	16.68	36.50
	6	NO_X_	0.949	1357.84	17.99	36.85
Testing	1	NO_X_	0.915	1141.55	15.53	33.79
	2	NO_X_	0.892	1463.17	18.60	38.25
	3	NO_X_	0.902	1403.59	20.39	37.46
	4	NO_X_	0.907	1277.21	18.33	35.74
	5	NO_X_	0.892	1471.76	17.43	38.36
	6	NO_X_	0.913	1241.92	19.37	35.24

**Fig 8:**
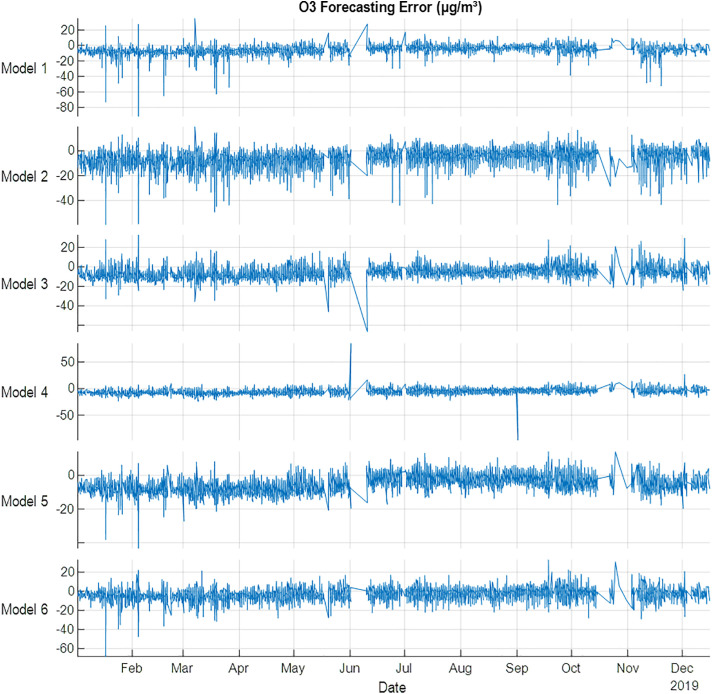
O3 error (µg/m³) forecasting errors for 2019 using NARX.

The forecasting of O_3_ is shown in Table 9 and Fig 8, the results showed that Model 5 is the best model for training and testing datasets. The results came in line with a correlation analysis in Table 3. The analysis shows that spring and Autumn are the most effective variables on O_3_ gas. Model 5 shows the lowest errors compared with other models. The results revealed that using a season effect has a great benefit in training all the models, as well as testing datasets.

**Table 9:** The performance of O_3_ gas prediction using training and testing datasets.

Dataset	Model	Gas	R^2^	MSE (µg/m³)²	MAE (µg/m³)	RMSE (µg/m³)
Training	1	O_3_	0.947	72.72	4.74	8.53
	2	O_3_	0.954	64.54	4.91	8.03
	3	O_3_	0.959	57.85	4.91	7.61
	4	O_3_	0.959	56.64	4.62	7.53
	5	O_3_	0.965	50.86	4.50	7.13
	6	O_3_	0.949	70.56	5.20	8.40
Testing	1	O_3_	0.946	65.04	6.32	8.06
	2	O_3_	0.928	69.67	6.25	8.35
	3	O_3_	0.948	57.66	6.30	7.59
	4	O_3_	0.959	53.79	6.15	7.33
	5	O_3_	0.963	47.69	5.75	6.91
	6	O_3_	0.941	42.82	4.99	6.54

**Fig 9:**
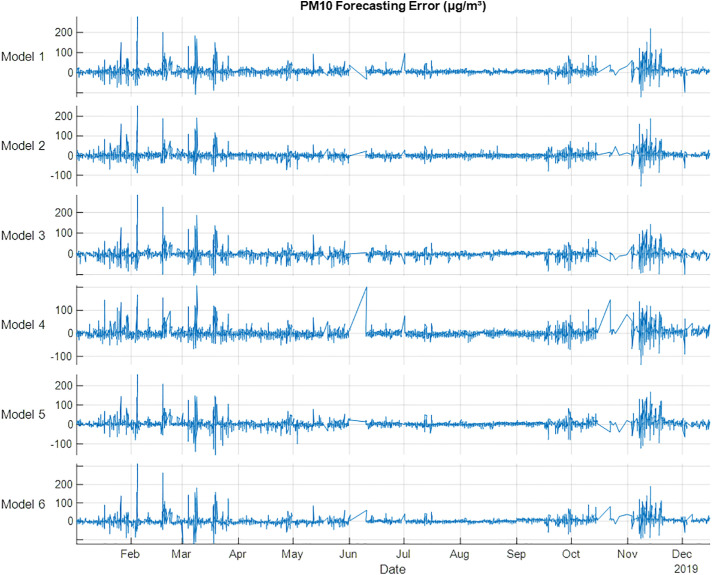
PM10 error (µg/m³) forecasting errors for 2019 using NARX.

**Fig 10:**
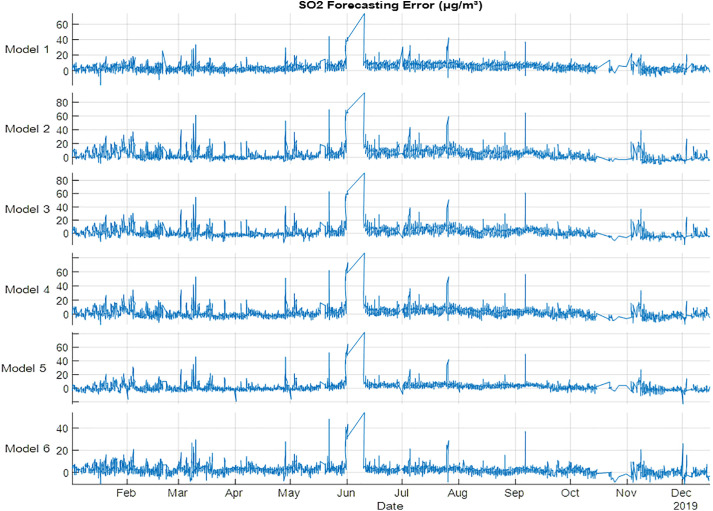
SO2 error (µg/m³) forecasting errors for 2019 using NARX.

PM_10_ forecasting results presented in Table 10 and Fig 9, Models 4 and 2 showed best performance for training and testing, respectively. The PM_10_ results showed that spring season has the best results compared with other models. The results indicate that using metrological variables with spring could retrieve the best performance metrics. SO_2_ forecasting has very bad results compared with NO, NO_2_, NO_X_, O_3_ and PM_10_, if Models 2–5 are used as shown in Table 11 and Fig 10. Training and testing datasets showed that Model 6 is the best model in improving performance metrics. The testing performance metrics are 0.888, 16, 3, 4 for R^2^, MSE, MAE and RMSE, respectively. The results indicate that using all the seasons could improve the performance of SO_2_ forecasting.

**Table 10:** The performance of PM_10_ gas prediction using training and testing datasets.

Dataset	Model	Gas	R^2^	MSE (µg/m³)²	MAE (µg/m³)	RMSE (µg/m³)
Training	1	PM_10_	0.943	669.14	13.09	25.87
	2	PM_10_	0.950	590.93	12.66	24.31
	3	PM_10_	0.947	631.69	12.81	25.13
	4	PM_10_	0.952	579.37	14.06	24.07
	5	PM_10_	0.944	664.06	13.17	25.77
	6	PM_10_	0.947	640.62	12.91	25.31
Testing	1	PM_10_	0.925	314.89	9.84	17.75
	2	PM_10_	0.927	256.98	8.79	16.03
	3	PM_10_	0.911	309.40	9.48	17.59
	4	PM_10_	0.919	291.38	10.18	17.07
	5	PM_10_	0.906	342.02	9.63	18.49
	6	PM_10_	0.915	303.95	9.05	17.43

**Table 11:** The performance of SO_2_ gas prediction using training and testing datasets.

Dataset	Model	Gas	R^2^	MSE (µg/m³)²	MAE (µg/m³)	RMSE (µg/m³)
Training	1	SO_2_	0.811	16.01	2.57	4.00
	2	SO_2_	0.683	24.80	2.95	4.98
	3	SO_2_	0.330	41.07	3.91	6.41
	4	SO_2_	0.550	32.20	3.44	5.67
	5	SO_2_	0.727	22.08	2.73	4.70
	6	SO_2_	0.803	16.59	2.52	4.07
Testing	1	SO_2_	0.824	34.60	4.37	5.88
	2	SO_2_	0.039	69.57	5.52	8.34
	3	SO_2_	0.338	48.53	4.62	6.97
	4	SO_2_	0.462	44.23	4.36	6.65
	5	SO_2_	0.756	28.08	3.46	5.30
	6	SO_2_	0.888	16.47	2.74	4.06

### 3.5. Discussion

Autumn, spring, summer and winter seasons have strong connections in increasing and decreasing the concentrations of PM_10_, SO_2_, NO, NO_X_, NO_2_ and O_3_ in air as shown in Table 12. Both training and testing datasets have different behaviors in forecasting. 50% of overall cases showed that Autumn and spring seasons are the dominant models in improving the performance metrics, while, without season effect model showed improvement for 20% of overall cases. In addition, summer-winter model, spring and autumn models showed 30% improvement in both datasets. Combining the results of training and testing datasets and eliminating the best results from training dataset. The best performance of PM_10_ is shown with metrological variable and spring season. SO_2_ and NO behaved very good with metrological variables with all seasons. NO_2_ and O_3_ showed a good concentration estimation with spring season and Autumn-spring seasons, respectively, where metrological variables are used as input with the seasons. NO_X_ has no improvement with any season and only using metrological variables are affected in NO_X_ forecasting. The results of PM_10_ and NO_2_ come in line with the feature selection method, where SO_2_, NO, NO_X_ and O_3_ are not in line with feature selection. This indicated that feature selection is appropriate for PM_10_ and NO_2_ only. Furthermore, the results obtained in this study are consistent with previous research conducted globally. For example, studies such as Zhang et al. [40] and Li et al. [41] confirmed that incorporating meteorological features significantly improves the forecasting accuracy of pollutants such as NO₂ and PM₁₀. These studies align with our findings where meteorological inputs and seasonal dummy variables led to higher R² values and lower errors for testing datasets. Moreover, similar approaches were validated in arid environments by Ahmed et al. [42], reinforcing the adaptability of machine learning models across different geographical contexts. Our work extends these findings by integrating a comprehensive seasonal representation, which further enhances the models’ generalization capabilities across different pollutants.

**Table 12:** The best model for each gas using training and testing datasets.

Dataset	Gas	R^2^	Best Model	Description
Training	PM_10_	0.952	4	Autumn Spring
	SO_2_	0.811	1	Without Season effect
	NO	0.950	5	Autumn Spring
	NO_2_	0.959	5	Autumn Spring
	NO_X_	0.951	5	Autumn Spring
	O_3_	0.965	5	Autumn Spring
Testing	PM_10_	0.927	2	Spring
	SO_2_	0.888	6	All seasons
	NO	0.901	6	All seasons
	NO_2_	0.973	3	Autumn
	NO_X_	0.915	1	Without Season effect
	O_3_	0.963	5	Autumn Spring

## 4. Conclusion

This work proposes a methodology of using feature selection to find the most effective season(s) on each air pollutant including PM_10_, SO_2_, NO, NO_2_, NO_X_ and O_3_. Based on the results of feature selection, 6 models are proposed, time series predictor NARX method is used to build a forecasting model using hourly training dataset between 2015 and 2018, where hourly testing dataset is used for validating the developed models. The dataset is adopted from Esenyurt, Istanbul. The main findings of this study can be summarized as follows:

- This paper is one of the few studies that considered the effect of season through feature selection method.
- It was found that Autumn and spring season are the most effective seasons on the concentration of NO_2_ and O3 gases, where spring season and autumn season are effective on PM_10_ and NO_X_, respectively. SO_2_ and NO gases, on the other hand, have impact with all seasons.
- NARX model is the most effective and accurate model to build prediction models for different gases’ concentration.
- All the prediction models that are used to construct different pollutants showed very good results except for SO_2_, the results were bad compared with other models.

This paper considered only one of the most polluted sites in Istanbul, Esenyurt and further sites needed to be conducted to validate different sites and different gases. Although the proposed forecasting models achieved strong performance, several limitations must be acknowledged. First, the models were trained and tested using data from a single geographic region, which may limit the generalizability of the results to other areas with different climatic or emission characteristics. Second, the study focused on a predefined set of pollutants and meteorological variables without incorporating potential influencing factors such as population density, industrial activity, or traffic flow. To address these limitations, future work should consider extending the dataset across multiple years and locations to enhance temporal and spatial generalizability. Integrating additional environmental and socio-economic variables may further improve predictive accuracy. Moreover, evaluating model performance using uncertainty quantification techniques and applying explainable AI (XAI) methods can offer deeper insights into the decision-making process of the models and increase transparency for real-world applications.

## Supporting information

S1 Dataset Excel file containing hourly air pollutant concentrations and meteorological variables collected in Esenyurt, Istanbul, Turkey (2015–2019, 37,645 records). The dataset was obtained from the National Air Quality Monitoring Network, Ministry of Environment, Urbanization, and Climate Change of Turkey, and is publicly accessible at https://www.havaizleme.gov.tr. (XLSX)
